# A Reappraisal of Saphenous Vein Grafting

**DOI:** 10.4103/0256-4947.75781

**Published:** 2011

**Authors:** Shi-Min Yuan, Hua Jing

**Affiliations:** From the School of Clinical Medicine, Nanjing University, Jinling Hospital, Department of Cardiothoracic Surgery, Nanjing, Jiangsu, China

## Abstract

Autologous saphenous vein grafting has been broadly used as a bypass conduit, interposition graft, and patch graft in a variety of operations in cardiac, thoracic, neurovascular, general vascular, vascular access, and urology surgeries, since they are superior to prosthetic veins. Modified saphenous vein grafts (SVG), including spiral and cylindrical grafts, and vein cuffs or patches, are employed in vascular revascularization to satisfy the large size of the receipt vessels or to obtain a better patency. A loop SVG helps flap survival in a muscle flap transfer in plastic and reconstructive surgery. For dialysis or transfusion purposes, a straight or loop arteriovenous fistula created in the forearm or the thigh with an SVG has acceptable patency. The saphenous vein has even been used as a stent cover to minimize the potential complications of standard angioplasty technique. However, the use of saphenous vein grafting is now largely diminished in treating cerebrovascular disorders, superior vena cava syndrome, and visceral revascularization due to the introduction of angioplasty and stenting techniques. The SVG remains the preferable biomaterial in coronary artery bypass, coronary ostioplasty, free flap transfer, and surgical treatment of Peyronie disease. Implications associated with saphenous vein grafting in vascular access surgery for the purpose of dialysis and chemotherapy are considerable. Vascular cuffs and patches have been developed as an important and effective means of enhancing the patency rates of the grafts by linking the synthetic material to the receipt vessel. In addition, saphenous veins can be a cell source for tissue engineering. We review the versatile roles that saphenous vein grafting has played as well as its current status in therapy.

Saphenous vein (SV) grafting has been broadly employed in a variety of operations including cardiac, thoracic, neurovascular, general vascular, vascular access, and urologic surgeries as a bypass conduit, interposition graft, and patch graft, with acceptable results, comparable to or better than for prosthetic materials.^1^,^2^ A modified SV graft (SVG) conduit such as a spiral SVG,^3^ a cylindrical SVG,^4^,^5^ or vein cuffs and patches,^6^ sometimes became a necessity for large recipient vessels, or to obtain a potentially good patency. Nowadays, an SVG is the preferable biomaterial in coronary artery bypass, especially for the right coronary system,^7^ coronary aneurysm or a rupture treated with SVG-covered stents,^8^,^9^ free flap transfer,^10^ and surgical treatment of Peyronie disease.^11^ However, the availability of new flexible intravascular stents, allowing access even to tortuous vessels, provides a new therapeutic approach for patients with vascular problems in diverse specialties. There have been significant declines in the use of SV grafting for the treatment of coronary ostioplasty,^12^ cerebrovascular disorders,^13^ superior vena cava syndrome,^14^ and visceral revascularization^15^ due to the introduction of angioplasty and stenting techniques. The percutaneous transluminal angioplasty and stenting, however, may develop complications or recurrent symptoms, which may eventually warrant surgical intervention.^15^ Surgical repair and percutaneous transluminal angioplasty become complementary and adopted in combination in selected patients. This review describes the versatile roles that SV grafting has played as well as the current use of this biomaterial.

## Roles for the Saphenous Vein Graft

### Vascular reconstruction of the extremities

Autologous SV grafting is popularly used in vascular surgery as a bypass graft, for the relief of hand and forearm ischemia,^16^ reconstruction of the axillary artery,^17^ or the brachial artery in an upper extremity,^18^ and femoropopliteal, femorotibial,^19^ plantar or lateral tarsal, tibioperoneal, and dorsalis pedis artery bypasses^20^ in a lower extremity. A femoropopliteal and femorotibial SVG bypass on 594 patients rendered a 5-year cumulative patency rate of 39.5% versus 64.9%.^19^ Comparing the results of 568 primary infrageniculate bypass procedures using SV grafting, polytetrafluoroethylene (PTFE), and PTFE-SVG, the 5-year limb salvage rate was 80% for composite grafts and 88% for SVGs. The primary and secondary patency and limb salvage rate for PTFE grafts was 24%, 31% and 40%, respectively.^21^ Arterial reconstruction of vessels of the foot and ankle using SV grafting as well as the arm vein for the management of extensive tibial and peroneal occlusive disease and patent pedal arteries showed 5.7% deaths and 4.2% graft failures within 30 days. Cumulative primary and secondary patency was 79.0% and 81.6% at 36 months, and limb salvage was 87.5% at 36 months.^20^

Palma and Esperon^22^ originally described a crossover femorofemoral bypass with an autologous SV grafting for the treatment of femoro-iliac venous occlusion, which was subsequently termed the Palma operation. This procedure is generally indicated for postphlebitis syndromes,^23^ postthrombotic syndrome,^24^ venous injury,^25^ pelvic tumor,^26^ and others. Menyhei et al^27^ reported a remarkable patency rate of 29/42 (69%) and excellent long-term results with the Palma operation performed for chronic venous insufficiency caused by unilateral iliac vein occlusion. A more recent report on the clinical results of five patients undergoing the Palma operation showed 4 patients with good patency, for whom the surgical indications were secondary to severe suprapubic and scrotal varicosities in 3, symptomatic pain and swelling in 1, and acute severe deep vein thrombosis in 1.^28^

**Figure 1:**
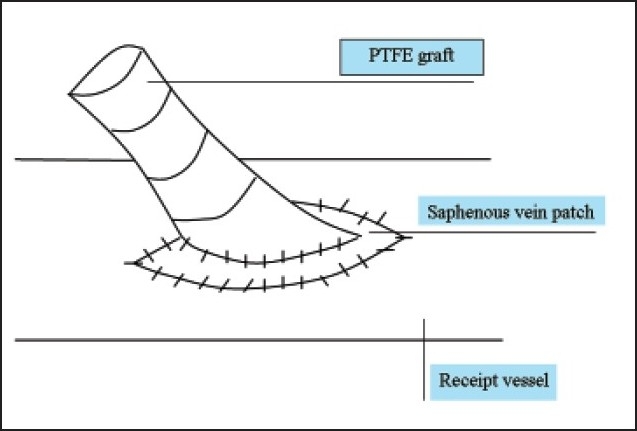
Linton patch.

**Figure 2:**
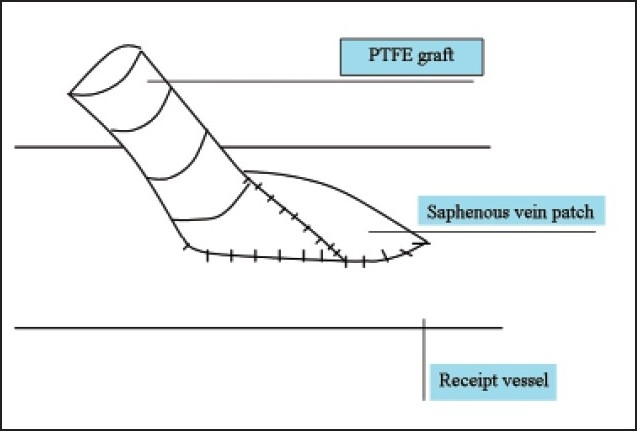
Taylor patch.

**Figure 3:**
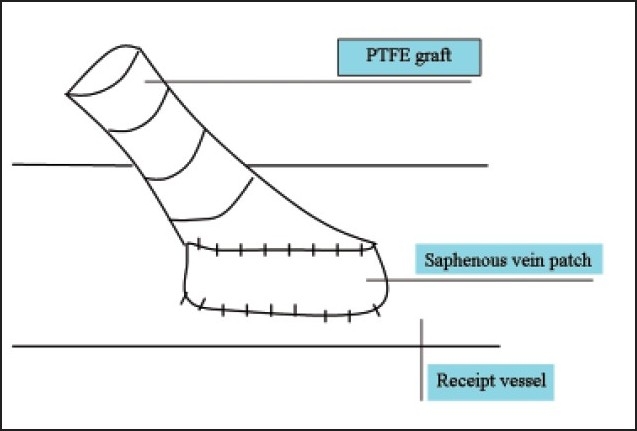
Miller cuff.

**Figure 4:**
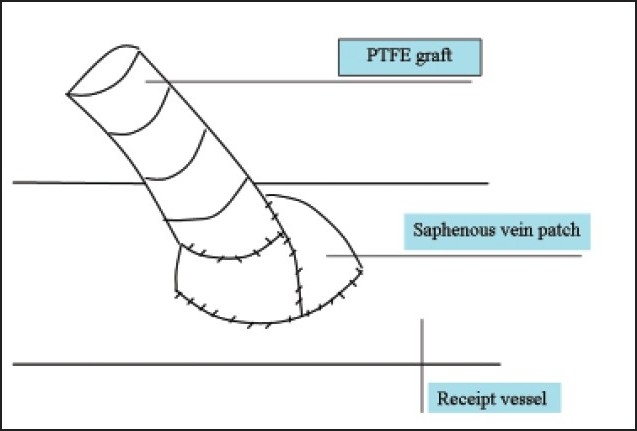
Vein boot.

Compliance mismatch between the graft and the recipient artery along with hemodynamic factors constitute the major causes of graft failure associated with thrombosis and the development of subintimal hyperplasia at the anastomotic site.^29^ In an attempt to obtain better patency, several vein patches and cuffs were developed by incorporating a segment of vein between the graft and the receipt vessel (**Figures 1–4**). Seigman^30^ suggested linking a Dacron tube to an artery with an interposed cylinder vein segment. Miller et al^31^ introduced this technique, which was later termed the Miller vein cuff, into clinical use as to build up the connection between rigid PTFE graft and the friable crural arteries, as a result, with higher patency rates. A multicenter randomized prospective study on 133 vein cuff and 128 uncuffed bypasses showed there was no differences in patency between cuff and non-cuff groups in the above-knee bypasses, but the vein cuff resulted in a better patency rate at 12 months, and a 20% higher limb salvage in the blow-knee patients at 24 months.^32^ Taylor patch is a modification to the conventional operative technique, involving a vein patch covering the elliptical defect between the anterior surface of the graft and the distal artery.^33^ Taylor et al^34^ obtained 5-year patency rates of 71% for popliteal and 54% for infrapopliteal grafts, respectively. Linton’s patch is a vein patch sutured to the artery, and a proximal venotomy is made on the patch and the PTFE material is sutured to the proximal venous tissue.^34^ The St. Mary’s boot (or vein collar) technique utilizes a similar arteriotomy and venous patch is modified into a collar shape and is sutured to arteriotomy.^35^

### Visceral revascularization

Arterial reconstruction with an autologous SVG patch or conduit is a method of choice for the surgical treatment of hepatic artery aneurysm.^36^,^37^ Successful hepatic vein reconstruction,^38^ or hepatic venoplasty^39^ was also performed by the use of an SVG in patients with hepatic malignancies. Reconstruction of the hepatic artery, hepatic vein, or portal vein in orthotopic liver transplant was usually performed as a standard procedure, using either an autologous, or cryopreserved third-party donor’s, or same donor’s SVG.^40^–^42^ Reconstruction of the celiac circulation in patients undergoing radical pancreaticoduodenectomy was often accompanied by an SVG bypass to relieve celiac artery occlusion of a congenital or secondary etiology.^43^,^44^

The repair of a long bile duct defect in a left hepatectomy for hepatocellular carcinoma may resort to a saphenous vein patch.^45^ Successful repair of iatrogenic common bile duct injuries has been achieved by SVGs in two patients with cystic duct avulsion, in one patient whose duct was split by a balloon catheter, and in one patient where a segment of the duct was resected. The grafts remained patent at a 5-year follow-up.^46^

SV grafting was also employed in visceral revascularization for variceal hemorrhage, hepatic cirrhosis, and acute mesenteric ischemia.^47^–^49^ Deen et al50 evaluated an autologous SVG anastomosed to the peritoneum in the management of patients with resistant ascites, 70% of whom did not require paracentesis any more.

### Aortorenal bypass

Aortorenal bypass was a standard technique for revascularization of the kidney with a compromised arterial circulation, such as in renovascular disease with severe hypertension,^51^ renal artery aneurysms,^52^,^53^ and renal artery dissection.^54^ The indications for an aortorenal bypass with a branched SVG were renovascular disease extending to two or more arterial branches, or having fibrous dysplasia, astherosclerosis, saccular aneurysm or stenosing disease involving multiple main renal arteries. The branched SVG was created by end-to-side anastomosis of a sidearm to the main graft.^55^ The search for a site of origin for renal artery bypass grafting other than the inclusion aorta has resulted in a variety of regimens, including use of the splenic, hepatic, gastroduodenal, and superior mesenteric arteries and even retrograde bypass grafts originating from the iliac artery.^56^

Aortorenal bypass was not only indicated for renovascular hypertension, renal failure due to stenotic arterial lesions, but also for acute anuria. Cole and Rabin^57^ performed a hepato-right renal arterial bypass with a reversed SVG in a patient with acute anuria 16 days after admission. The amount of urine excretion returned to normal, and the serum creatinine level stabilized without dialysis.

When the aorta cannot be used for a standard renal bypass because of a previous aortic operation, severe degenerative atherosclerosis or complete aortic thrombosis, a unilateral (hepatic) or bilateral (hepatic or splenic) visceral bypass should be considered. A right hepatorenal artery SVG bypass and a splenorenal artery anastomosis on the left plus a hepatorenal artery SVG bypass on the right side were performed in two patients with degenerative abdominal aorta, respectively.^58^

### Other operations

**Figure 5:**
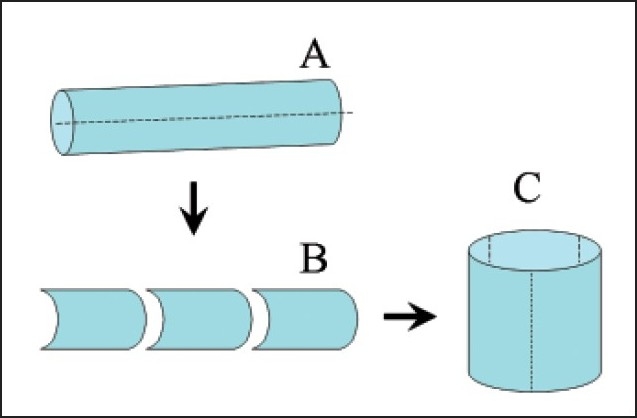
Cylindrical vein graft.

SV grafting has even been useful when remodeled into a cylinder configuration for a size-match purpose for the construction of either jugular or portal veins. Urayama et al4 and Sakamoto et al5 respectively utilized such remodeled SVGs where the saphenous vein was split longitudinally and sutured side-to-side (**Figure 5**) with good results. SV grafting added its versatility as catheter conduit for arterial infusion chemotherapy to treat hepatocellular carcinomas and metastatic liver cancer after hepatectomy or in unresectable patients with satisfactory perfusion.^59^

## Cardiac Surgery

### Coronary artery bypass

It is well know that a reversed autologous SVG remains a preferable conduit for a coronary artery bypass, with an independent graft (a single graft with two anastomoses as an outflow and an inflow) being the classic fashion. The sequential graft (a graft with one inflow anastomosis and more than one outflow anastomoses in single or different receipt coronary arteries) was introduced in an aim to decrease the number of anastomoses, shorten the operative time, and improve graft patency.^60^ A long SVG was once proposed to complete circular sequential bypasses with as more as five distal anastomoses, to posterior descending right coronary artery, two marginal branches, diagonal branch, and a left anterior descending artery.^61^ Naturally formed Y-branches 2 cm in length made it possible to perform Y-grafts, sequential grafts, or a combination of the two, or more complex configurations for quadruple and quintuple bypasses by a single SVG.^62^ Nonreversed valvotomized SVG has additionally been recommended for coronary artery bypass.^63^ In the operation, the femoral end of the vein is attached to the aorta and the pedal end is attached to the coronary artery, and it assured a large proximal anastomosis and satisfactory patency rate. Besides, composite arterial grafts (a complex graft configuration composed of at least two segments of one artery or two different arteries) were developed under the requirement of complete arterial revascularization.^64^ Modified bypass configurations as composite mixed arterovenous grafts (a composite graft composed of the artery and vein, usually internal mammary artery and SVG) were also developed in case arterial graft could not reach the anastomosing site.^65^

**Figure 6:**
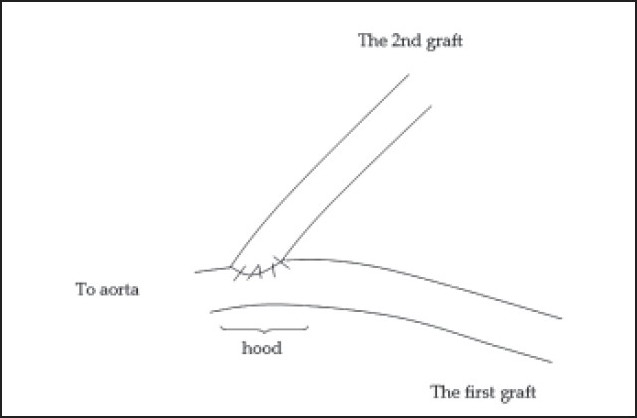
Vein hood.

An SVG can also function as a hood of a second graft (**Figure 6**). The indication for anastomosing a second vein or arterial graft onto a vein graft hood is an inadequate length of the second graft or the avoidance of proximal anastomoses on an atheromatous ascending aorta.^66^ John^67^ suggested the second surgical option for the mismatch between aortotomy and SVG size is to disconnect the vein from the aortotomy, and then to anastomose it in an end-to-side manner to another SVG that has already been joined to the aorta.

### Coronary ostioplasty

Coronary ostioplasty with an autologous saphenous vein patch is an alternative approach to standard bypass for the patients with isolated coronary ostial stenosis.^68^ Dihmis and Hutter^69^ modified the technique of left coronary angioplasty by insertion of a gusset of long SVG into the left main coronary artery and adjacent aorta. This technique was then extended to patients with atherosclerotic or nonatherosclerotic coronary artery disease.^70^ Jegaden et al^71^ extended this surgical technique into the coronary trunk angioplasty in 12 patients, the first two of which were performed with saphenous vein patch. All procedures were successful. Surgical ostioplasty should be considered in the treatment of patients who have isolated ostial stenosis but no distal coronary disease. Careful patient selection seems to be a prerequisite for surgical success.^68^

### Aneurysmorrhaphy

Aneurysmorrhaphy with SVG patch reconstruction is a preferred approach for the treatment of coronary artery aneurysms, thereby maintaining the antegrade flow, preserving the important perforator branches, and avoiding bypass grafting to the distal segment.^72^–^74^ Postoperative coronary angiography revealed disappearance of the aneurysm and no stenosis of the repaired coronary artery.^74^

### Other procedures

SV grafting has additional uses in cardiac surgery. Axillary cannulation can be achieved by placing the arterial cannula into an SVG that had been anastomosed end-to-side to the axillary artery. This provides a natural, inexpensive, and more hemostatic alternative to the use of prosthetic grafts.^75^ A homologeous SVG can be used as a conduit to replace the malignancy-invaded inferior vena cava,^76^ to create an aortopulmonary communications,^77^ or to construct modified Blalock-Taussig shunts^78^,^79^ in patients with cyanotic congenital heart disease who have satisfactory patency.

## Interventional Cardiology

### Obliteration of coronary thrombus or aneurysm

The implantation of covered stents has emerged as a strategy for treatment when traditional conservative approaches, such as prolonged balloon inflation and reversal of anticoagulation, fails.^80^ Like other harvested vascular segments, including autologous cephalic vein and antecubital vein, an autologous SVG was used as a cover for the stents to obliterate coronary artery thrombus,^81^ aneurysms of the coronary artery,^9^ or SVG,^82^ for immediate exclusion of the aneurysm as well as thrombus and maintaining patency compared to conventional stents. The experimental studies have shown a beneficial effect with covered stents on biocompatibility, endothelialization and vascular injury.^83^

## Thoracic Surgery

### Surgical treatment of superior vena cava syndrome

Saphenojugular anastomotic technique was adopted as an effective treatment for malignant or benign superior vena cava syndrome since the early 1960s, with promising results.^84^,^85^ To avoid graft kinking and compression, Panneton et al^86^ modified the saphenojugular bypass by tunneling an externally supported ePTFE graft subcutaneously to protect the SVG, in that the ipsilateral SVG was turned cephalic and tunneled through an ePTFE graft and anastomosed end-to-end in a spatulated manner with the contralateral SVG tunneled down from the internal jugular vein. The graft patency was promising as confirmed by duplex ultrasound scanning.

**Figure 7:**
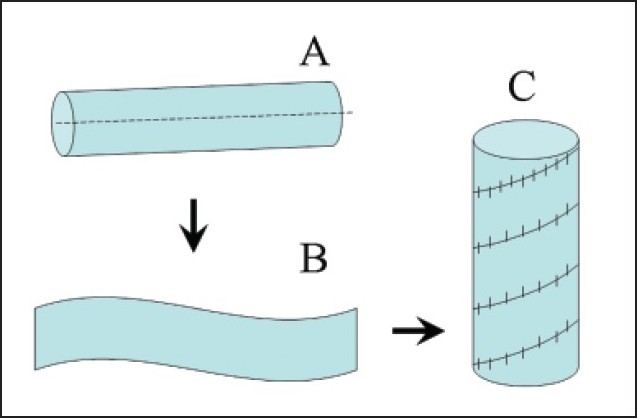
Spiral vein graft.

In 1962, Benvenuto et al^87^ proposed a spiral vein graft for replacement of the superior vena cava. Doty and Baker^88^ extended this technique for reconstruction of the occluded superior vena cava in 1976. A composite spiral vein graft was constructed from the patient’s own saphenous vein, split longitudinally and wrapped around a stent in spiral fashion (**Figure 7**). The edges of the vein were sutured together to form a large conduit ranging in diameter from 9.5 to 15.0 mm.^89^ Dotty et al^89^ reported that seven of nine grafts remain patent for up to 15 years and all but one patient was free of superior vena caval syndrome. A spiral vein graft showed favorable clinical outcomes for caval replacement as other autologous conduits. The disadvantage may include tedious and time-consuming construction of the spiral vein, and potential thrombosis that might be caused by the long suture line. In addition, reversed autogenous SVGs could be used in patients with superior vena caval obstruction secondary to mediastinal fibrosis.^90^

## Neurosurgery

### Cerebral revascularization

The use of an SVG for a bypass or reconstructive purpose was a preferable treatment of choice in neurosurgery for the management of extracranial atherosclerotic disease, extra- and intracranial aneurysms, and tumors involving the carotid artery at the skull base or cervical regions.^13^ Cerebral revascularization was established in three ways: a superficial temporal artery to middle cerebral artery bypass; a long interposition SVG between the carotid artery in the neck and the branches of the middle cerebral artery, or a short SVG from the intrapetrous to the supraclinoid carotid.^13^

In 1969, Yasargil performed the first extracranial-to-intracranial bypass with ligation of the middle cerebral artery for the treatment of a complex cerebral aneurysm. This surgical technique was then employed widely in the treatment of giant intracranial aneurysms.^91^ When the middle cerebral artery is not suitable for intracranial anastomosis, the supraclinoid internal carotid artery can be a recipient vessel. Cervical-to-petrous internal carotid artery anastomosis in cases of upper cervical or petrous internal carotid artery aneurysms or tumors, vertebral artery (extracranial)-to-vertebral artery (intracranial), vertebral artery-to-posterior cerebral artery, and internal carotid artery-to-basilar artery bypass were also established by using an SVG.^92^

A long vein bypass graft used for the treatment of a giant aneurysm was first described by Iwabuchi et al^93^ in 1979. The long SVG was made popular by Sundt et al^94^ for atheromatous disease and for giant aneurysms involving the anterior and posterior circulation. In a series of 20 patients with internal carotid artery aneurysms unsuitable for clipping or coiling, long venous bypasses were interposed between the internal carotid artery at the neck and the intrapetrous carotid, from the internal carotid artery at the neck to a branch of the middle cerebral artery, or from the external carotid artery to a branch of the middle cerebral artery. The surgical results were admirable with a 95% patency rate at a follow-up of 1-12 years (mean 3.7 years).^95^ An SVG was generally one of the bypass grafts in the tandem bypass (a long extracranial-to-intracranial bypass with two grafts of different materials).^96^

A short graft may be placed between the petrous portion of the internal carotid artery and the intradural portion of internal carotid artery or middle cerebral artery. Short vein grafts have some disadvantages compared to long ones in poor exposition of the petrous carotid artery, sacrifice of the great petrosal nerve, technical difficulty, and discontinuation of blood flow of the internal carotid artery for 30-60 minutes. A long saphenous vein graft proved more effective and safe in providing high-flow bypass in the anterior circulation.^97^

Initial experiences with interposed SVGs for ischemic and traumatic occlusion of the internal carotid artery and intracranial aneurysm obtained encouraging results with an overall patency rate of 80% at a 12-month follow-up.^98^ In cases of distal vertebral artery disease, the SVG was placed either from the C2 transverse foramen to the intradural portion of the vertebral artery, or from the extradural C1 portion to the intradural artery beyond the posterior inferior cerebellar artery.^99^ Morgan et al^100^ reported 57 interposition SVG bypasses between the common carotid artery and the intracranial internal carotid artery in 55 patients, with which early graft occlusion was 5%.

The literature on cerebrovascular venous reconstruction is rare. Steiger et al^101^ reported a 48-year-old female with who developed a hemongiopericytic meningioma involving the middle third of the superior sagittal sinus, which was replaced with a 6 cm SVG, harvested from the thigh to select a segment with a side branch for anastomosis with the left Rolandic vein. The sagittal sinus was replaced with the SVG. The left Rolandic vein was sutured to the SVG side branch, and the right Rolandic vein was anastomosed end-to-side to the lateral wall of the sagittal sinus behind the graft. The patient recovered uncomplicated after operation.

## Plastic and Reconstructive Surgery

### Free flap transfer

Free flap transfer for the treatment of tissue defect caused by trauma,^102^ tumor resection,^103^ irradiation therapy,^104^ or a chronic lumbosacral wound,^105^ anywhere in the head and neck, trunk, or extremities, was usually accomplished by an SVG. Salibian et al^104^ described a 2-stage transfer of a latissimus dorsi musculocutaneous flap for coverage of a radiation ulcer of the sacral area. Nahai and Hagerty^106^ performed the same procedure for the similar case by using SVGs in one stage. Meanwhile, a short SVG was taken into use in free flap transfer for defects of the lower extremity,^107^ traumatic tissue defects of the trunk,^102^ and the wounds caused by tumor resection.^103^ Chang et al^108^ anastomosed two flaps to a single autologous SVG for both a primary arterial conduit in an end-to-end fashion and a secondary end-to-side recipient site in the microsurgical reconstruction of a complicated head and neck defect, which survived perfectly. An SVG arteriovenous loop in a free-flap transfer for the treatment of a chronic lumbosacral wound,^105^ and cryopreserved SVG was used on an emergency basis for lower extremity reconstruction^109^ were respectively reported. Citrin and Dasmahapatra^110^ advocated using the spiral SVG bypass of the internal jugular vein when bilateral radical neck dissections were performed with the sacrifice of both internal jugular veins. They performed such procedures in six patients without significant swelling or facial edema in any patient.

## Urology

### Lengthening shortened penis caused by Peyronie disease

The penile deep dorsal vein, inferior tibial vein, and saphenous vein are the grafts of choice in the surgical treatment of shortened penis caused by Peyronie disease.^111^ The plaque incision with saphenous vein grafting, known as a Lue procedure is now the standard operation for treating penile shortening due to Peyronie disease.^112^ The technique involves an H-shaped tunical incision to release the contracture followed by defect repair with the use of an assembled SVG segments.^113^ Clinical investigations have shown that an excellent or satisfactory result was obtained in 92% to 93% of patients,^114^,^115^ and the penis was completely straightened in 82% to 96% at a mean follow-up of 12-18 months.^112^,^115^,^116^ The application of a W-shaped SVG, which was molded according to the tunica defect, was associated with a straightened penis in 87.5% of the patients at a mean follow-up of 13 months.^117^ For patients with severe penile shortening due to Peyronie disease, circumferential grafting was performed using SVG.^118^

## Vascular Access Surgery

In 1972, Lawton and Sharzer^119^ started the use of SV grafting in the construction of an arteriovenous fistula for patients needing prolonged hemodialysis. Construction of subcutenous arteriovenous fistulae for hemodialysis with autologous SVGs used to be done in five ways 5: straight radial artery-cephalic vein, loop brachial artery-cephalic vein, straight brachial artery-auxiliary vein, straight axillary artery-basilic vein, and femoral artery-saphenous vein stump fistulas. It was noted that the loop arteriovenous fistula in the forearm was inferior to the straight in terms of short- and long-term patency rates.^120^ Cimochowski et al^121^ reported their successful experience with the use of a spiral vein in vascular access in a single patient who had undergone 16 prior access operations with no more adequate access for dialysis. In this case, a spiral saphenous vein graft was constructed from the left saphenous vein and used as a straight arterial conduit in the groin as the sole dialysitic route for the next consecutive 750 dialysis procedures over nearly 6 years without any complication. Gagne et al^122^ used a Tyrell vein collar at the venous anastomosis of forearm loop arteriovenous grafts in 17 patients undergoing hemodialysis, but noted a premature graft failure with a 9-month primary patency of 17% compared to 80% for the control group with a standard end-to-side graft-vein anastomosis.

The vascular access for chemotherapy was always wrist radiocephalic or elbow cephalic, basilica or medial cubital vein to branchial fistula. Wobbes et al^123^ suggested, when suitable vessels were unavailable in the upper extremities, an arteriovenous fistula be performed in the inguinal region using the long SVG. In 100 consecutive patients with various malignancies, 142 operations were performed to establish an arteriovenous fistula giving vascular access for chemotherapy. Radiocephalic fistula was established in 88 operations on 76 patients and functioned well in 64%. In 13 patients whose arms offered no alternative possibility, 15 long SVGs were implanted in the inguinal region. Guba et al^124^ successfully administered chemotherapeutic agents, blood products and hyperalimentation solutions and recurrent diabetic ketoacidosis via vascular access procedures in 13 patients. Vascular access for chemotherapy by an autologous SVG fistula was also reported by Levey et al,^125^ who created in infants and children with malignancy, a loop fistula from the SVG to the superficial femoral artery. Such access could provide with rapid dilute chemicals and last as long as 3 years.

## Tissue Engineering

The saphenous vein can be a source of tissue engineering. Studies have shown that that cells isolated from the saphenous veins, or from veins and arteries of the umbilical cord might be feasible cell sources for tissue engineering of heart valve for the pulmonary position.^126^

## Discussion

When an autologous saphenous vein is unavailable, a homologous saphenous vein under different preservation methods, such as frozen, denatured, lyophilized, cryopreserved, and fresh, can be an alternative in creating vascular access. Preserved vein homografts tolerate repeated puncture by large dialysis needles. Similarly, when satisfactory autologous SVGs are not available, cryopreserved homologous SVGs, either cryopreserved or denatured, can be an alternative conduit to the autologous ones in coronary artery bypass,^127^ construction of aortopulmonary communication,^77^ a modified Blalock-Taussig shunt,^78^,^79^ and complex limb-salvage procedures.^128^,^129^ Some authors^127^,^130^,^131^ have suggested that use of such conduits should be limited due to poor patency. Despite various types of stents that have been used to treat atherosclerotic stenoses of coronary, renal, and superficial femoral arteries, open surgery is still the treatment of choice when the angioplasty fails and the patient develops recurrent symptoms.^15^ Straight spiral SVG remains the conduit of choice for surgical reconstruction, with results superior to those with bifurcated veins and ePTFE. Endovascular treatment is effective over the short term, with a frequent need for repeat interventions.^14^

The use of an SVG bypass in neurosurgery has decreased largely because of improved endovascular therapies in many circumstances. The “gold standard” for the treatment of giant aneurysms remains surgical clipping. Nevertheless, a small but consistent number of SVG bypass procedures will be required for the treatment of complex cerebrovascular disease.^13^ Arterialized venous free flap transfers with the long saphenous vein will be favorable in the reconstruction of major arteries of the injured skin and soft tissues.^132^ Generally, synthetic materials are no longer used in grafting procedures in Peyronie surgery because of their antigenicity and inappropriate functional properties. Small intestinal submucosa may be associated with a high rate of operative failure and complications. SV grafting is the preferred autologous graft with acceptable outcomes.^133^ Arteriovenous grafts remain an important vascular access option for dialysis, and interventions to prevent progression of stenosis are being explored.^134^ Recent data indicate that the majority of patients on hemodialysis in the United States have prosthetic graft fistulas. The most frequent complications of prosthetic graft fistulas are thrombosis and stenosis.^135^ Endovascular interventions have replaced surgical repair as the primary treatment of the failing or thrombosed vascular access. Angioplasty is a fast, easy, and safe procedure that can extend the patency of a hemodialysis graft.^136^

In conclusion, SV grafting plays an important role as a material superior to the prosthesis in bypass grafting, interposition conduit, patch repair, loop creation, vein cuff, stent cover, catheter route in many circumstances, and has shown excellent outcomes as evidenced by the patency rates of the autologeous graft. Percutaneous transluminal angioplasty and stenting techniques have largely substituted vascular repair and reconstruction procedures with admirable results. The use of SV grafting has dwindled with the introduction of percutaneous transluminal angioplasty and stenting techniques, but in indicated cases the use of SV grafting can be unavoidable. Hence it is each surgeon’s responsibility to husband every centimeter of the SVG during harvestment, as we have seen even the varicose SVG put into use.
